# Spinal Metastasis from Supratentorial Glioblastoma: A Registry-Based Case Series and a Review of the Literature

**DOI:** 10.3390/cancers17182979

**Published:** 2025-09-12

**Authors:** Arthur Chak Kai Lau, Desiree Ka-ka Wong, Justin Chun Him Cheung, Candice H. W. Lam, Myron Chak Him Wong, Jason Chak Yan Li, Danny T. M. Chan, Herbert H. F. Loong, Michael W. Y. Lee, Tony K. T. Chan, Jason M. K. Ho, Ka-Man Cheung, Teresa P. K. Tse, Joyce S. W. Chow, Aya El-Helali, Peter Y. M. Woo

**Affiliations:** 1 Department of Neurosurgery, Prince of Wales Hospital, Hong Kong; 2 Otto Wong Brain Tumour Centre, The Chinese University of Hong Kong, Hong Kong; 3 Department of Clinical Oncology, Queen Elizabeth Hospital, Hong Kong; 4 Department of Neurosurgery, Queen Elizabeth Hospital, Hong Kong; 5 Department of Clinical Oncology, The Chinese University of Hong Kong, Hong Kong; 6 Department of Neurosurgery, Pamela Youde Nethersole Eastern Hospital, Hong Kong; leewym@ha.org.hk; 7 Department of Neurosurgery, Princess Margaret Hospital, Kwai Chung, Hong Kong; 8 Department of Neurosurgery, Tuen Mun Hospital, Hong Kong; 9 Department of Clinical Oncology, The University of Hong Kong, Hong Kong; ahelali@hku.hk

**Keywords:** glioblastoma, spinal metastasis, overall survival, magnetic resonance imaging, radiotherapy, spinal surgery

## Abstract

**Background**: Spinal metastasis is a rare complication of supratentorial glioblastoma. We report the clinical features and prognosis of this phenomenon and review the relevant literature. **Methods**: This is a territory-wide, multicentre, retrospective review using data from the Hong Kong High-grade Glioma Registry from 2006 to 2023. Data of consecutive adult patients diagnosed with supratentorial glioblastoma and spinal metastasis were extracted and analyzed. **Results**: Among the 1342 patients with supratentorial glioblastoma, 15 were diagnosed to have spinal metastasis (1.1%). The median time to spinal metastasis from the initial diagnosis of glioblastoma was 38.7 weeks (IQR: 15.1–57.6). Multi-level spinal involvement was present in 60% (9/15) of patients. Neither the topographical location of the tumor in relation to the subventricular zone, extent of resection, occurrence of intraoperative ventricular entry, nor *methylguanine methyltransferase* (*MGMT*) promoter methylation status predicted the time to spinal metastasis. The median overall survival was 44.1 weeks (IQR: 29.9–80.2), and the median post-spinal metastasis survival was 12.6 weeks (IQR: 5.0–15.0). Two-thirds of patients received spinal radiotherapy, 26.7% had systemic therapy (chemotherapy, targeted therapy, and/or immunotherapy), and 13.3% underwent surgical spinal decompression. No significant survival improvement was observed among patients who received spinal radiotherapy (HR: 0.61; 95% CI: 0.17–2.23) or systemic therapy (HR: 0.94; 95% CI: 0.20–4.39). **Conclusions**: This case series illustrates the management practices and clinical course of glioblastoma patients with spinal metastasis. No treatment modality was proven to be superior. Treatment remains largely palliative and should be tailored on an individual basis.

## Simple Summary

Glioblastoma is the most common primary brain malignancy among adults and carries a poor prognosis. In rare circumstances, glioblastoma can metastasize to the spinal cord, resulting in severe focal neurological deficits, including paralysis and incontinence. We report the clinical presentation, treatment, and outcomes of 15 patients with this rare phenomenon, identified from a territory-wide brain tumor registry in Hong Kong. Treatment for glioblastoma spinal metastasis may include a combination of spinal surgery, radiotherapy, and/or systemic therapies. Reviewing the current medical literature, no particular treatment has been proven to be effective in conferring longer survival, but they may serve to alleviate symptoms and reduce further neurological damage. Despite its rarity, spinal metastasis of glioblastoma should not be overlooked, and clinical vigilance is required in order to provide timely treatment.

## 1. Introduction

Glioblastoma is the most common primary malignant brain tumor in adults and has an incidence of 1–5 per 100,000 [1,2]. Standard of care consists of maximal safe tumor resection followed by temozolomide (TMZ) chemoradiotherapy (CRT) [3]. Despite multimodal treatment, the prognosis of glioblastoma patients remains poor, with an overall survival (OS) of 10–14 months [2,4,5]. While most patients invariably experience tumor recurrence, up to 90–95% are focal, i.e., within 2 cm of the resection cavity, and distant recurrences typically remain intracranial [6,7,8,9]. Extracranial metastasis to the spinal cord is a rare complication, and there is no consensus on their treatment. We present a series of 15 patients diagnosed with supratentorial glioblastoma with spinal cord metastasis. We describe the clinical characteristics and management of this rare phenomenon with a review of the biomedical literature.

## 2. Methods

This was a retrospective territory-wide multicenter review of adult patients (≥18 years) with histologically confirmed supratentorial glioblastoma with documented evidence of spinal metastasis between 1 January 2006 and 30 December 2023. All patients received maximal safe resection of their primary tumor across all seven neurosurgical centers in Hong Kong. The original tumor diagnosis was made in accordance with the World Health Organization (WHO) Classification of Central Nervous System Tumors, 4th Edition [10]. This study was approved by the institutional review board of the Hospital Authority (HA) of Hong Kong (reference number: KC/KE-18-0262/ER-4). All patients were identified from the Hong Kong High-grade Glioma Registry (HK-HGG registry), a centralized prospective repository of consecutive patients with histologically proven high-grade glioma treated by the city’s public healthcare system. In Hong Kong, universal public healthcare is provided by the HA, a statutory government body that delivers care for over 90% of inpatient bed-days in the city. From the HK-HGG registry, we manually reviewed the medical records of all glioblastoma patients (*n* = 1342) within the study period. The following inclusion criteria were adopted: (1) International Classification of Diseases, 9th Edition (ICD-9) codes 198.3 (secondary malignant neoplasm of brain and spinal cord), 198.4 (secondary malignant neoplasm of other parts of the nervous system), 192.2 (malignant neoplasm of spinal cord), and C72.0 (malignant neoplasm of spinal cord); (2) clinical documentation of spinal metastasis in electronic patient records; (3) radiological evidence of leptomeningeal, intramedullary, or drop dissemination to the spinal cord; and/or (4) lumbar puncture cerebrospinal fluid (CSF) cytology reports confirming the presence of tumor cells. Patients who had primary brainstem, cerebellar, or spinal gliomas, those with Isocitrate dehydrogenase-1 (*IDH*-1) mutant gliomas, or who only had a clinical suspicion of spinal dissemination without supporting radiological or pathological evidence were excluded.

Collected data were broadly classified into patient-related, tumor-related, and treatment-related categories. Patient-related data included demographics and preoperative Karnofsky performance status (KPS) upon presentation of their brain tumor, and clinical presentation upon diagnosis of spinal metastasis. Tumor-related data included the location of the supratentorial glioblastoma, its relation to the subventricular zone (SVZ), methylation status of the *methylguanine methyltransferase* (*MGMT*) promoter, and the location of spinal involvement. The SVZ was defined as a 3–5 mm region bordering the lateral wall of the lateral ventricle [11,12]. This was ascertained by reviewing preoperative magnetic resonance imaging (MRI) that demonstrated the gadolinium contrast-enhancing tumor’s involvement of the SVZ. Treatment-related data included the extent of resection (EOR) of the primary supratentorial tumor during the index operation, whether there was entry into the ventricular system during resection, and the oncologic treatments for the primary and subsequent metastatic spinal tumor. Kaplan–Meier curves were used to plot time-to-outcome data, including time to spinal metastasis, post-spinal metastasis survival, and overall survival (OS). OS was defined as the duration from the date of the index resection to the date of death. The data-cutoff date for this study was 31 December 2023. Data are reported using standard biostatistical methods. Patients without a documented date of death were censored at the date of last clinical follow-up. Following univariate analysis, a multivariate Cox regression analysis was performed to adjust for the effects of established OS prognostic factors for glioblastoma patients. A *p*-value < 0.05 was considered statistically significant. All statistical analyses and plots were generated using the Statistical Package for the Social Sciences software version 21.0 (SPSS Inc., Chicago, IL, USA).

## 3. Results

Fifteen glioblastoma patients with spinal metastasis were identified among our review of 1342 individuals with supratentorial glioblastoma (1.11%). The mean age at diagnosis of the primary supratentorial glioblastoma was 40.7 years (±14.1 years; range: 18–63 years), and the female-to-male ratio was 1:1.5. Preoperative Karnofsky Performance Scale (KPS) was ≥80 in two-thirds (10/15) of patients. The majority of patients (12/15; 80.0%) were ethnic Chinese. *MGMT* promoter methylation was present in five tumors (41.7%). The primary tumor was predominantly located in the temporal lobe in 60% (9/15) of patients, and half involved the SVZ (8/15; 53.3%).

Gross total resection (GTR) of the supratentorial glioblastoma was achieved at the index operation for seven patients (46.7%). Ventricular entry (VE) occurred during primary tumor resection in 80% (12/15) of patients, and seven (47.6%) subsequently required ventriculoperitoneal shunting for hydrocephalus. Following the index surgery, eleven patients (73.3%) proceeded with TMZ CRT. Nine patients (60%) received adjuvant-phase TMZ therapy with a mean number of 6.8 cycles (±SD 4.4 cycles). Of the four patients who were not offered adjuvant TMZ CRT, two patients had *BRAF*^V600E^ mutated-tumors (13.3%, 2/15) and both had spinal metastases upon presentation. They were hence offered palliative targeted therapy instead of TMZ CRT. One patient (P14) received a series of systemic agents including TMZ, combination dabrafenib (BRAF inhibitor) with trametinib (MEK inhibitor), and subsequently nivolumab. She survived 31.4 weeks and 28.7 weeks from her diagnosis of supratentorial glioblastoma and spinal metastases, respectively. Another patient (P12) received combination dabrafenib with trametinib and succumbed 17.1 weeks after being diagnosed with supratentorial glioblastoma and 15.4 weeks since spinal metastases (Table 1).

**Figure 1:**
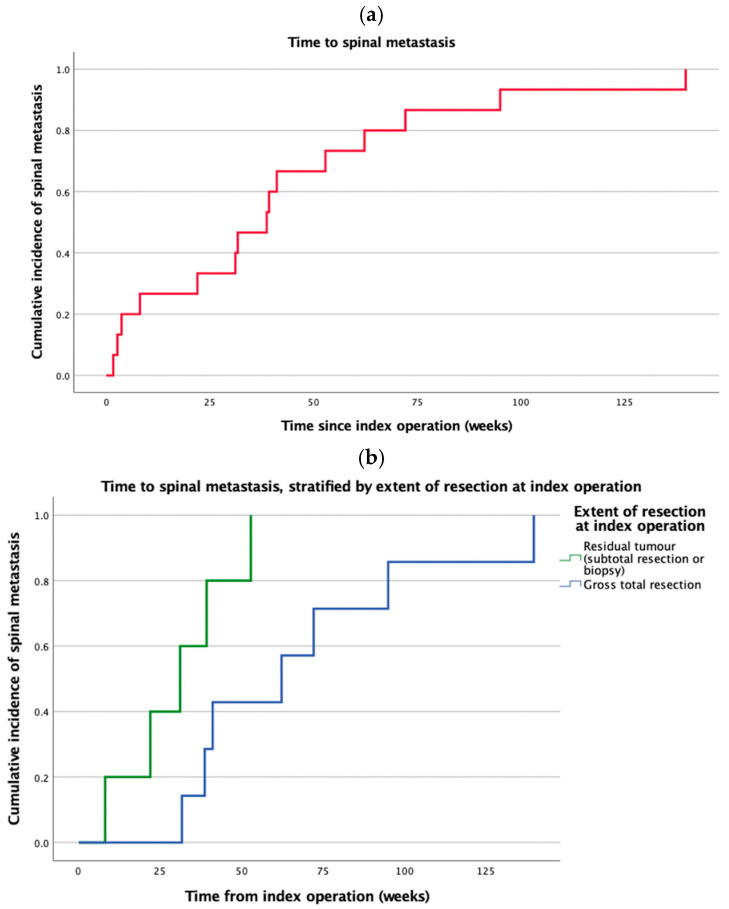

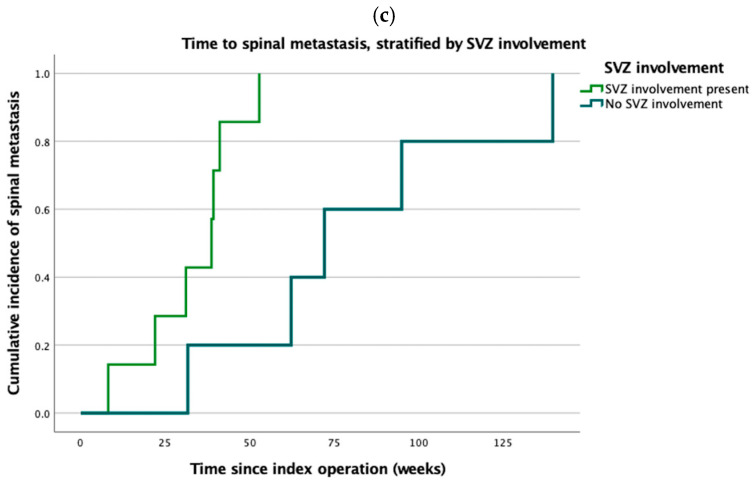
Time to spinal metastasis. (**a**) Cumulative incidence of spinal metastasis over time; (**b**) stratified by extent of resection at index operation; (**c**) stratified by SVZ involvement.

Spinal metastasis was present at the time of the diagnosis of supratentorial glioblastoma (i.e., within one month of the index operation) in three patients (20.0%) (Table S1). Overall, the median time to spinal metastasis was 38.7 weeks (IQR: 15.1–57.6 weeks) (Figure 1a). Eight patients (53.3%) had associated intracranial tumor recurrence at a median of 40.6 weeks (IQR: 27.9–58.6 weeks). Univariate analysis of the 12 patients with spinal metastasis upon recurrence demonstrated that the presence of residual tumor after the index resection (HR 4.63, 95% CI: 1.08–19.9, *p* = 0.04; Figure 1b) and SVZ involvement (HR 10.1, 95% CI: 1.18–85.6, *p* = 0.03; Figure 1c) were associated with earlier spinal metastasis. *MGMT* promoter methylation (HR 0.60, 95% CI: 0.16–2.18, *p* = 0.43) or intraoperative VE (HR 0.63, 95% CI: 0.13–3.18, *p* = 0.58) were not found to be associated with earlier spinal metastasis. After adjusting for age, sex, and KPS, the association between SVZ involvement and earlier spinal metastasis was significant (adjusted HR 8.3, 95% CI: 0.6–116.5, *p* = 0.12). No association between EOR and time to spinal metastasis was demonstrated (HR 1.42, 95% CI: 0.1–17.6, *p* = 0.78).

**Figure 2:**
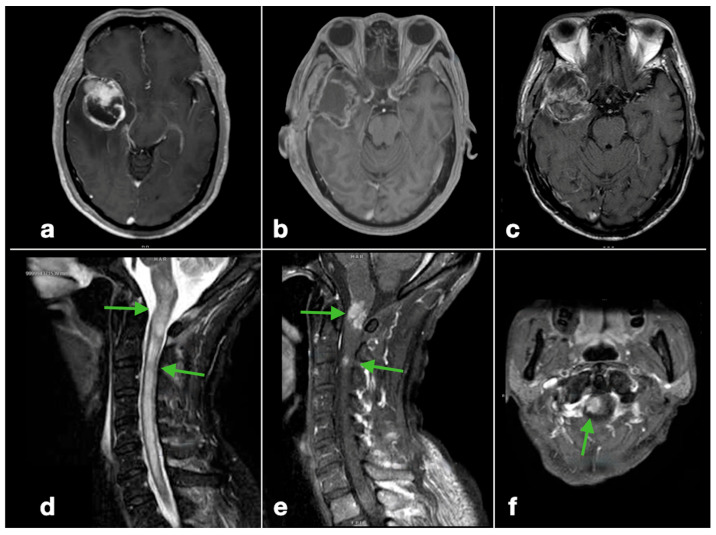
Radiological features of glioblastoma spinal metastases (Illustrative patient 1): a 58-year-old woman with right temporal glioblastoma involving the SVZ ((**a**), gadolinium-contrast–enhanced T1-weighted MRI, axial) underwent near-total resection ((**b**), postoperative day 1 contrast-enhanced T1W MRI, axial). After TMZ concomitant chemoradiotherapy, there was focal tumor recurrence eight months after diagnosis ((**c**), axial), along with metachronous spinal tumor deposits at the cervicomedullary junction and C2/3 level ((**d**), T2W cervical spine MRI, sagittal; (**e**), contrast-enhanced T1W cervical spine MRI, sagittal; (**f**), axial C2/3 level). (The green arrows denote the metastatic spinal lesions).

**Figure 3:**
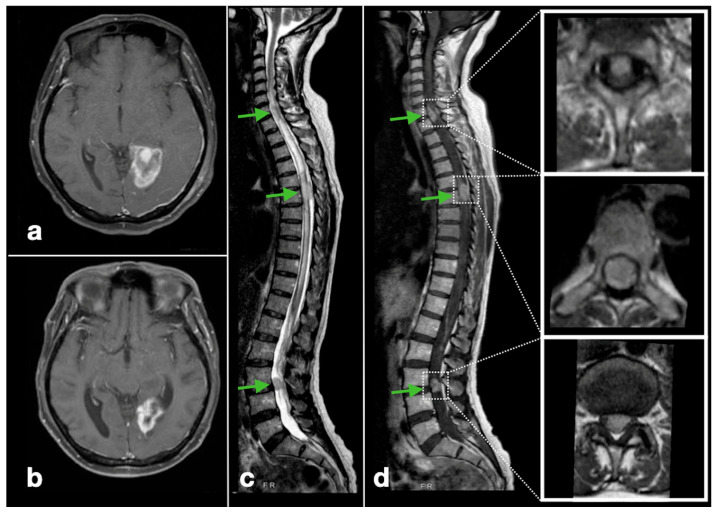
Radiological features of glioblastoma spinal metastases (Illustrative patient 2): a 64-year-old man with a left lingula gyrus glioblastoma involving the SVZ (**a**) gadolinium-contrast enhanced T1-weighted MRI, axial) underwent near-total resection. After TMZ concomitant chemoradiotherapy, there was focal tumor recurrence ten months after diagnosis ((**b**), axial) with metachronous spinal tumor deposits at the C7/T1, T5/6 and L3/L4 levels ((**c**), T2W whole-spine MRI, sagittal; (**d**), contrast-enhanced T1W whole-spine MRI, sagittal, and corresponding spinal level involvement in the inset, axial). (The green arrows denote the metastatic spinal lesions).

**Figure 4:**
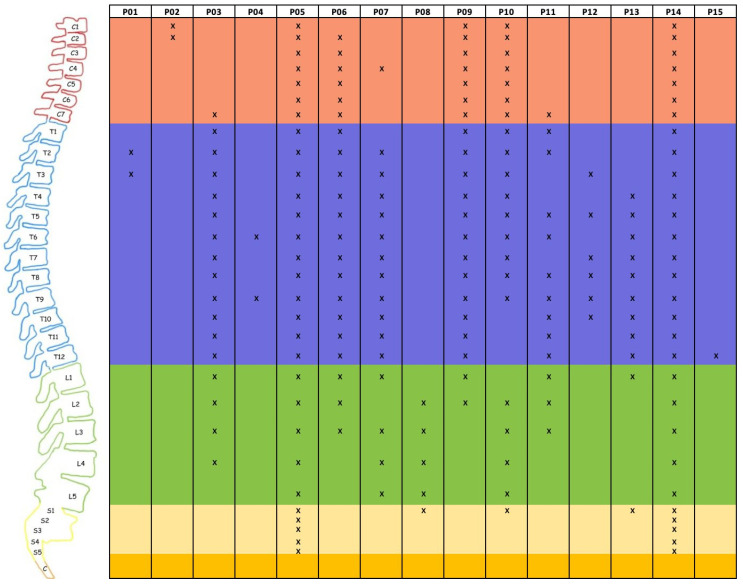
Distribution and extent of glioblastoma metastatic spinal lesions. (“X” denotes the levels of spinal cord involvement as detected on spinal MRI).

Spinal involvement was symptomatic in 14 patients (93.3%). The majority presented with paraparesis (9/15, 60.0%) and/or paraesthesia with neurogenic bladder symptoms (6, 40.0%). Five patients (30.0%) had either neck pain or back pain (Table 2). Diagnosis was confirmed radiologically in all 15 patients using spinal gadolinium contrast-enhanced MRI (Figure 2 and Figure 3). More than half (60%) had multi-level involvement, with metastases involving two or more levels of the cervical, thoracic, lumbar, and/or sacral spine. The most commonly affected spinal levels were the thoracic spine, affecting 12 patients (80.0%), followed by the lumbar spine (10/15; 66.7%), cervical (9/15; 60.0%), and sacral regions (4/15; 26.7%) (Figure 4). Among lumbar cistern CSF studies collected from eight patients, five (62.5%) had positive tumor cytological findings.

**Figure 5:**
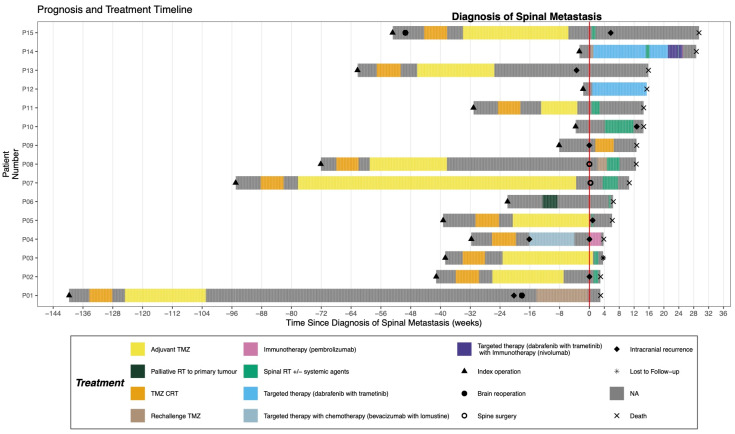
Swimmer plot depicting the disease course of the glioblastoma patients with spinal metastasis.

Most patients (12/15; 80%) received treatment directed at the spinal metastasis, including resection, radiotherapy, and/or systemic agents, either as monotherapy or combined multi-modal treatment (Figure 5). The majority of patients (10/15; 66.7%) underwent palliative fractionated spinal radiotherapy, and four (26.7%) were administered systemic oncologic therapy. The median biologically effective dose delivered using radiotherapy was 39.9 Gy (IQR: 31.3–41.8 Gy_8_; assuming an α/β ratio of 8 Gy [13]). Besides the two patients with *BRAF*^V600E^-mutated tumors who received BRAF/MEK targeted therapy (dabrafenib and trametinib), one patient continued with TMZ CRT, and another was prescribed pembrolizumab monotherapy.

**Figure 6:**
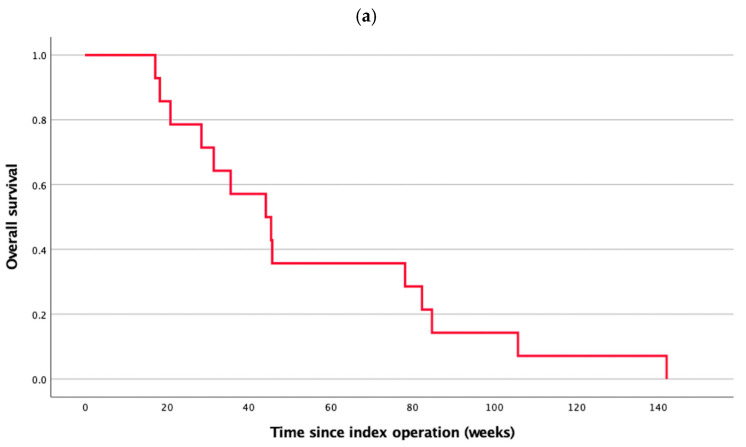

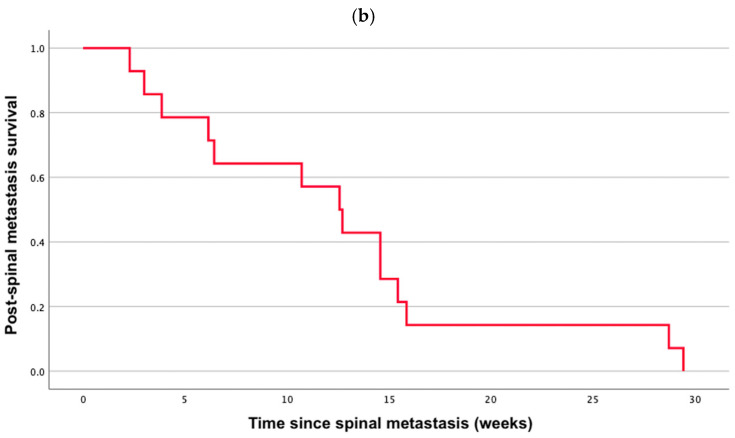
Survival outcomes glioblasatoma patients with spinal metastasis. (**a**) Overall survival; (**b**) post-spinal metastasis survival.

The median OS was 44.1 weeks (IQR: 29.9–80.2 weeks) (Figure 6a) and the median post-spinal metastasis survival was 12.6 weeks (IQR: 5.0–15.0 weeks) (Figure 6b). There was no demonstrable benefit in post-spinal metastasis survival from either RT (HR 0.74, 95% CI: 0.23–2.35, *p* = 0.61) or systemic therapy (HR 0.85, 95% CI: 0.26–2.80, and *p* = 0.79).

## 4. Discussion

Spinal metastasis is a rare and serious complication of glioblastoma. Since glioblastoma invariably carries a short survival, most patients likely succumb to the primary tumor before developing clinically evident spinal metastasis [14,15,16,17]. In spite of the rarity of this phenomenon, historical autopsy series detected evidence of spinal metastasis in up to 15–25% of patients with supratentorial glioblastoma [18,19,20]. In contrast to the postmortem observations, the incidence of clinically significant spinal metastasis has commonly been reported to be 1–2% [15,21,22,23,24]. Under-reporting is likely to exist, since spinal imaging is not routinely performed for asymptomatic patients, and spine-related symptoms may be masked by neurological deficits arising from the primary intracranial tumor. To estimate the incidence of asymptomatic spinal metastasis at initial diagnosis, Shibahara et al. performed routine spinal MRI screening in newly diagnosed glioblastoma patients, regardless of the presence of spine-related symptoms. Of the 87 patients who underwent screening spinal MRI, 11 patients (12.6%) showed radiological evidence of spinal dissemination. Only one of the 11 patients with spinal metastasis hadattributable limb weakness at diagnosis, whereas the remaining 10 patients had no spine related symptoms [22]. Nevertheless, with advancements in diagnostic radiology, such as the application of CSF-sensitivity imaging (e.g., post-contrast fluid-attenuated inversion recovery imaging (FLAIR) sequences), it is expected that spinal metastases could be detected more readily [25].

The clinical presentation of spinal metastases depends on the location and degree of spinal cord compression, such as neck or back pain, radicular pain, gait disturbance, paraplegia, paraesthesia, neurogenic bladder, bowel symptoms, and sexual dysfunction [14,15,21,23,24,26,27,28,29,30,31,32,33,34,35,36,37,38,39,40]. Therefore, detection of spinal involvement heavily relies on maintaining a high index of clinical suspicion. In our cohort, while the majority of the patients (60%) had multilevel involvement, the thoracic spine was the most commonly affected region (80%), followed by the lumbar spine and cervical spine, corroborating previous observations [15,23,41]. One postulation to explain this trend is that the thoracic cord is more sensitive to compressive lesions, given the relatively narrower spinal canal in this region [23].

MRI is the chief investigation for diagnosis of spinal metastasis but is usually not performed as a routine screening tool in real-world practice [17,23]. Alongside conventional contrast-enhanced imaging, inclusion of FLAIR sequences to the routine glioma MRI protocol has been advocated to improve the diagnostic accuracy for both intracranial and spinal leptomeningeal metastasis, particularly in patients with *IDH*-*1* wildtype astrocytoma [25,42]. The superior performance of FLAIR sequences in detecting leptomeningeal disease has been ascribed to its ability in suppressing normal leptomeningeal vasculature, allowing a clearer delineation of genuine lesions [25,43,44,45]. Lumbar CSF cytological examination for tumor cells is another investigation of choice. However, the sensitivity of CSF cytology is low, and only a small proportion of glioblastoma patients with spinal metastases demonstrate malignant or suspicious cells in CSF samples. The CSF cytology positivity rate has been reported as low as 0% in a case series of 14 glioblastoma patients with radiological evidence of spinal dissemination [14,22,46]. CSF protein levels and D-dimer levels have been investigated as potential surrogate biomarkers for spinal dissemination of glioblastoma, but larger-scale studies are required to support these findings [22,46].

The exact oncobiological mechanisms for spinal metastases remain elusive. It had been suggested that the risk of spinal metastasis increases with unmethylated *MGMT* promoter status, higher Ki-67 score, temporal lobe location, SVZ involvement, and multiple surgical resections [22,47,48,49,50,51]. The role of intraoperative VE as a risk factor for leptomeningeal dissemination has been debated. A meta-analysis of nine studies with high-grade gliomas observed a strong association between intraoperative VE and leptomeningeal dissemination (OR 3.9, 95% CI: 1.9–8.1, *p* = 0.0002), translating into shorter OS (HR 1.3, 95% CI: 1.1–1.5, *p* = 0.01) [49]. Subsequent data revealed that instead of intraoperative VE, SVZ tumor involvement was an independent risk factor for leptomeningeal spread (HR 1.9, 95% CI: 1.1–3.3, *p* = 0.01) and poorer OS (HR 1.9, 95% CI: 1.4–2.7, *p* < 0.001) [52]. Since VE is more commonly encountered during resections of SVZ-involving glioblastomas (VE in 66/114 of SVZ-involving tumors versus 19/118 of non-involving tumors), it was concluded that SVZ tumor involvement likely confounded previous observations regarding the importance of VE in leptomeningeal tumor dissemination and OS [52]. Similarly, a retrospective cohort study of 200 patients did not observe a higher rate of subsequent leptomeningeal disease in patients who had VE during primary glioblastoma resection (7.5% with VE *versus* 10.2% without VE, *p* = 0.57) [53].

A number of molecular and genomic alterations have been observed in glioblastomas with metastatic dissemination. Earlier case studies identified *PTEN* gene mutation, gains at 1p36 and 1q25 chromosomal regions, and a high MIB-1 labelling index to be prognostic of leptomeningeal dissemination [54,55]. Prior to the publication of the 5th WHO classification system for CNS tumors, which integrated *IDH* status to the molecular diagnostic criteria for glioblastoma, an *IDH*-wildtype status has also been associated with a higher risk of spinal metastasis [47,56]. More recently, stanniocalcin-1 (STC-1), a microRNA-regulated secreted glycoprotein with physiological functions in autocrine-paracrine signalling, has been recognised as a metastasis-promoting factor in various cancers, including glioblastoma, by activating PI3K/AKT and JNK signalling pathways [57]. A retrospective study of tumor tissues and CSF samples from 23 glioblastoma patients with metastatic dissemination to the spinal cord or medulla oblongata demonstrated significantly reduced expression levels of several STC-1-regulating microRNAs as well as increased STC-1 mRNA expression compared to those without metastasis, implicating a possible association for STC-1 in promoting glioblastoma CSF dissemination [58]. 

Despite neurosurgical resection, radiotherapy, and systemic therapy, the median survival following spinal metastases was only 12.6 weeks in our cohort. This short survival was similar to the median survival of 11.2 weeks in previous meta-analysis [23]. There is currently no standard therapeutic recommendation given the rarity of this phenomenon. A combination of radiotherapy, chemotherapy, targeted therapy, and immunotherapy was employed. We were unable to demonstrate a significant survival benefit from either RT or systemic therapy. A single-center retrospective study of 168 recurrent glioma patients with leptomeningeal spread, of which 29 patients had glioblastoma with spinal metastasis, also did not observe any significant OS difference between patients that received RT, chemotherapy, or antiangiogenic therapy [59].

Surgical resection is largely reserved for palliative purposes in selected patients, but it has not been shown to confer a survival benefit [14,15,17,23,28,29,30,32,34,35,36,39,60,61,62,63,64]. Experience with primary spinal high-grade glioma suggested that postoperative RT, rather than resection was associated with prolonged survival [65]. Since most patients with spinal tumor metastasis already have recurrent disease, delays in initiating second-line chemotherapy or RT after resective surgery for wound-healing reasons could inadvertently truncate their OS.

External beam radiotherapy is the most common treatment offered for glioblastoma spinal metastatic lesions [23]. A few case reports have described a modest effect of RT in preserving neurological function and alleviating pain in patients withspinal metastasis [29,34,35,66,67,68,69,70]. Nevertheless, no survival benefit was demonstrated with spinal RT [15,59]. There is also no evidence-based consensus on the optimal RT regimen and fractionation schedule. Data from QUANTEC (Quantitative Analyses of Normal Tissue Effects in the Clinic) have estimated that, under full-circumference irradiation to the spinal cord with conventional fractionation of 1.8–2 Gy/fraction, the risk of myelopathy is 0.03% at a maximal cord dose (D_MAX_) of 45 Gy, 0.2% at D_MAX_ of 50 Gy, <1% at D_MAX_ of 54 Gy, and <10% at D_MAX_ of 61 Gy [71]. With emerging knowledge on spinal cord tolerance, there has been growing interest in introducing stereotactic radiotherapy for primary spinal glioblastomas, allowing for the delivery of a higher biologically effective dose to optimise local tumor control and alleviate symptoms, while reducing the risk of radiation-induced myelopathy [72,73].

Existing clinical practice guidelines for recurrent glioblastomas such as from, the National Comprehensive Cancer Network (NCCN), the European Association of Neuro-Oncology (EANO), and the European Society for Medical Oncology (ESMO) recommended systemic therapy options including rechallenge with temozolomide, nitrosoureas, procarbazine-lomustine, bevacizumab, and regorafenib [74,75,76], but none have been proven to be superior [77]. Although bevacizumab, an anti-angiogenic vascular endothelial growth factor inhibitor, has been suggested for patients with leptomeningeal glioblastoma metastases [78,79].

There are several study limitations. The latest WHO classification adopts a multi-layer integrated approach to defining glioblastoma, integrating histological features with molecular signatures: *IDH-1* mutation, *EGFR* amplification, *TERT* promoter mutations, and the combined gain of chromosome 7 and loss of chromosome 10 [80]. However, this study consisted of a historical cohort spanning over 15 years, and it was not standard practice to perform the complete panel of molecular testing in previously. Second, the sample size of this series was not large enough to conclude or compare the effectiveness of various treatment modalities for spinal metastasis. Third, there was limited access to data regarding spinal metastasis-related symptom control and quality of life. Finally, there was no well-selected control cohort undergoing routine spinal MRI surveillance to delineate which patients were most at risk of developing subclinical metastasis. However, given the high morbidity and functional impact of this condition, real-world reviews of this distinct glioblastoma condition should be considered.

## 5. Conclusions

Spinal metastasis is a rare complication of supratentorial glioblastoma. No clear benefit in OS was observed for any specific therapy adopted for this condition, and a high level of vigilance is required when patients experience either neck pain, back pain, or spinal cord compression syndromes. Suurvival after the detection of spinal meastasis is short, but palliative treatment may be considered such as spinal decompressive surgery or RT. 

Future research should aim at identifying the subset of glioblastoma patients at risk of metastasis for opportunistic spinal image screening. With more frequent detection, adequately powered prospective clinical trials may then become feasible to investigate novel therapies for these patients.

## Figures and Tables

**Table 1:** Baseline patient, tumor and treatment characteristics of the included patients.

	Total (*n* = 15) (%)
***Patient and tumour characteristics***	
**Age**, years,	
mean ± SD (years)	40.7 ± 14.1
**Sex (%)**,	
Male,	9 (60.0)
Female	6 (40.0)
**Preoperative KPS** (%)	
≥80	10 (66.7)
≤70	5 (33.3)
**Location of tumour by lobe** (%)	
Frontal	2 (13.3)
Temporal	9 (60.0)
Parietal	3 (20.0)
Occipital	1 (6.7)
Central core *	3 (20.0)
**Subventricular zone involvement** (%)	
Yes	8 (53.3)
No	7 (46.7)
***IDH-1*** **mutation** (valid %)	
Wildtype	9 (100.0)
Not available	6
**p*****MGMT*** **promoter methylation** (valid %)	
Methylated	5 (41.67)
Unmethylated	7 (58.33)
Not available	3
***First line treatment for supratentorial glioblastoma***	
**Extent of resection** (%)	
Gross total resection	7 (46.7)
Subtotal resection	7 (46.7)
Biopsy	1 (6.7)
**Intraoperative ventricular entry** (%)	
Yes	12 (80.0)
No/not applicable	3 (20.0)
**First line treatment received** (%)	
Surgery	15 (100.0)
Concurrent chemoradiotherapy (CCRT)	11 (73.3)
Adjuvant-phase temozolomide therapy	9 (60)
***Disease recurrence and survival***	
**Intracranial disease recurrence (%)**	8 (53.3)
**Time to intracranial disease recurrence**,	40.6
median (IQR), weeks	(27.9-58.6)
**Overall survival from glioblastoma diagnosis**, median (IQR), weeks	44.1 (29.9–80.2)

* The central core comprises of the insula, basal ganglia, and thalamus.

**Table 2:** Characteristics of glioblasatoma patients with spinal metastasis.

	Total (*n* = 15) (%)
***Spinal metastasis characteristics***	
**Timing of spinal metastasis**	
Upon presentation	3 (20.0%)
Upon disease recurrence	12 (80.0%)
**Time to spinal metastasis from the initial diagnosis of glioblastoma**, median (IQR), weeks	38.7 (15.1–57.6)
**Method of spinal metastasis diagnosis**	
Contrast-enhanced MRI spine (%)	15 (100.0)
Positive lumbar puncture CSF cytology (% of the patient who had cytological studies for CSF)	5 (62.5)
**Presenting symptoms** Limb weakness Sensory disturbances Sphincter symptoms (acute retention of urine/urinary incontinence) Neck pain/back pain Cranial nerve deficit Asymptomatic	9 (64.3) 6 (42.9) 6 (42.9) 5 (35.7) 1 (7.1) 1
**Location/extent of spinal metastasis** (%)	
Cervical-spine only	1 (6.7)
Thoracic-spine only	4 (26.7)
Lumbar-spine only	1 (6.7)
Multi-level involvement †	9 (60.0)
***Treatment for the spinal metastatic lesion ‡***	
**Laminectomy for resection**	2 (13.3)
**Spinal radiotherapy**	10 (66.7)
**Systemic treatment** §	4 (26.7%)
- Chemotherapy (TMZ) ¶	2
- Targeted therapy (Dabrafenib with trametinib)	2
- Immunotherapy (Pembrolizumab or nivolumab)	2
***Post-spinal metastasis survival***	
**Post-spinal metastasis survival**, median (IQR), weeks	12.6 (5.0–15.0)

† Multi-level involvement was defined as involvement in two or more spinal levels. ‡ Of the ten patients who received spinal RT, two were also given systemic treatment. Of the five patients who did not receive spinal RT, one patient was treated with immunotherapy monotherapy and another were treated with targeted treatment alone. § One patient sequentially received chemotherapy, targeted therapy, and immunotherapy. ¶ One patient had TMZ as part of the standard CRT for newly diagnosed glioblastoma, as the spinal metastases was diagnosed before the start of CRT.

## Data Availability

The datasets used and/or analyzed during the current study are available from the corresponding author upon reasonable request.

## Supplementary Materials

The following supporting information can be downloaded at: https://www.mdpi.com/article/10.3390/cancers17182979/s1. Table S1: Characteristics of the three patients with spinal metastasis at the time of the diagnosis (i.e. <1 month of the index operation).

## Abbreviations

The following abbreviations are used in this manuscript:

CI	Confidence interval
CRT	Chemoradiotherapy
CSF	Cerebrospinal fluid
EOR	Extent of resection
FLAIR	Fluid-attenuated inversion recovery imaging
GTR	Gross total resection
HR	Hazard ratio
*IDH-1*	Isocitrate dehydrogenase-1;
IMRT	Intensity-modulated radiotherapy
IQR	Interquartile range
KPS	Karnofsky performance status
MRI	Magnetic resonance imaging
OS	Overall survival
*pMGMT*	Methylguanine-methyltransferase promoter
RT	Radiotherapy
SD	Standard deviation
SVZ	Subventricular zone
TMZ	Temozolomide
VE	Ventricular entry

## References

[1] Woo P.Y.M., Yau S., Lam T.C., Pu J.K.S., Li L.F., Lui L.C.Y., Chan D.T.M., Loong H.H.F., Lee M.W.Y., Yeung R. (2023). Patterns of care and survival of Chinese glioblastoma patients in the temozolomide era: A Hong Kong population-level analysis over a 14-year period. Neuro-Oncol. Pract..

[2] Ostrom Q.T., Price M., Neff C., Cioffi G., Waite K.A., Kruchko C., Barnholtz-Sloan J.S. (2023). CBTRUS Statistical Report: Primary Brain and Other Central Nervous System Tumors Diagnosed in the United States in 2016–2020. Neuro-Oncology.

[3] Stupp R., Mason W.P., Bent M.J.v.d., Weller M., Fisher B., Taphoorn M.J.B., Belanger K., Brandes A.A., Marosi C., Bogdahn U. (2005). Radiotherapy plus Concomitant and Adjuvant Temozolomide for Glioblastoma. N. Engl. J. Med..

[4] Johnson D.R., O’Neill B.P. (2012). Glioblastoma survival in the United States before and during the temozolomide era. J. Neuro-Oncol..

[5] Brodbelt A., Greenberg D., Winters T., Williams M., Vernon S., Collins V.P. (2015). Glioblastoma in England: 2007–2011. Eur. J. Cancer.

[6] De Bonis P., Anile C., Pompucci A., Fiorentino A., Balducci M., Chiesa S., Lauriola L., Maira G., Mangiola A. (2013). The influence of surgery on recurrence pattern of glioblastoma. Clin. Neurol. Neurosurg..

[7] Rapp M., Baernreuther J., Turowski B., Steiger H.J., Sabel M., Kamp M.A. (2017). Recurrence Pattern Analysis of Primary Glioblastoma. World Neurosurg..

[8] Langhans M., Popp I., Grosu A.L., Shusharina N., Binder H., Baltas D., Bortfeld T. (2023). Recurrence analysis of glioblastoma cases based on distance and dose information. Radiother. Oncol..

[9] Woo P.Y.M., Law T.H.P., Lee K.K.Y., Chow J.S.W., Li L.F., Lau S.S.N., Chan T.K.T., Ho J.M.K., Lee M.W.Y., Chan D.T.M. (2024). Repeat resection for recurrent glioblastoma in the temozolomide era: A real-world multi-centre study. Br. J. Neurosurg..

[10] Louis D.N., Ohgaki H., Wiestler O.D., Cavenee W.K., Burger P.C., Jouvet A., Scheithauer B.W., Kleihues P. (2007). The 2007 WHO classification of tumours of the central nervous system. Acta Neuropathol..

[11] Quiñones-Hinojosa A., Sanai N., Soriano-Navarro M., Gonzalez-Perez O., Mirzadeh Z., Gil-Perotin S., Romero-Rodriguez R., Berger M.S., Garcia-Verdugo J.M., Alvarez-Buylla A. (2006). Cellular composition and cytoarchitecture of the adult human subventricular zone: A niche of neural stem cells. J. Comp. Neurol..

[12] Beiriger J., Habib A., Jovanovich N., Kodavali C.V., Edwards L., Amankulor N., Zinn P.O. (2022). The Subventricular Zone in Glioblastoma: Genesis, Maintenance, and Modeling. Front. Oncol..

[13] Pedicini P., Fiorentino A., Simeon V., Tini P., Chiumento C., Pirtoli L., Salvatore M., Storto G. (2014). Clinical radiobiology of glioblastoma multiforme: Estimation of tumor control probability from various radiotherapy fractionation schemes. Strahlenther. Onkol..

[14] Birbilis T.A., Matis G.K., Eleftheriadis S.G., Theodoropoulou E.N., Sivridis E. (2010). Spinal metastasis of glioblastoma multiforme: An uncommon suspect?. Spine.

[15] Lawton C.D., Nagasawa D.T., Yang I., Fessler R.G., Smith Z.A. (2012). Leptomeningeal spinal metastases from glioblastoma multiforme: Treatment and management of an uncommon manifestation of disease. J. Neurosurg. Spine.

[16] Vollmer K., Pantazis G., Añon J., Roelcke U., Schwyzer L. (2019). Spinal Metastases of Supratentorial Glioblastoma with Primitive Neuronal Component. World Neurosurg. X.

[17] Amelot A., Terrier L.M., Cognacq G., Jecko V., Marlier B., Seizeur R., Emery E., Bauchet L., Roualdes V., Voirin J. (2023). Natural history of spinal cord metastasis from brain glioblastomas. J. Neuro-Oncol..

[18] Erlich S.S., Davis R.L. (1978). Spinal subarachnoid metastasis from primary intracranial glioblastoma multiforme. Cancer.

[19] Onda K., Tanaka R., Takahashi H., Takeda N., Ikuta F. (1989). Cerebral glioblastoma with cerebrospinal fluid dissemination: A clinicopathological study of 14 cases examined by complete autopsy. Neurosurgery.

[20] Yung W.A., Horten B.C., Shapiro W.R. (1980). Meningeal gliomatosis: A review of 12 cases. Ann. Neurol..

[21] Tinchon A., Oberndorfer S., Marosi C., Rudà R., Sax C., Calabek B., Grisold W. (2012). Malignant spinal cord compression in cerebral glioblastoma multiforme: A multicenter case series and review of the literature. J. Neuro-Oncol..

[22] Shibahara I., Saito R., Osada Y., Kanamori M., Sonoda Y., Kumabe T., Mugikura S., Watanabe M., Tominaga T. (2019). Incidence of initial spinal metastasis in glioblastoma patients and the importance of spinal screening using MRI. J. Neuro-Oncol..

[23] Wright C.H., Wright J., Onyewadume L., Raghavan A., Lapite I., Casco-Zuleta A., Lagman C., Sajatovic M., Hodges T.R. (2019). Diagnosis, treatment, and survival in spinal dissemination of primary intracranial glioblastoma: Systematic literature review. J. Neurosurg. Spine.

[24] Vertosick F.T., Selker R.G. (1990). Brain stem and spinal metastases of supratentorial glioblastoma multiforme: A clinical series. Neurosurgery.

[25] Park Y.W., Jang G., Kim S.B., Choi K., Han K., Shin N.-Y., Ahn S.S., Chang J.H., Kim S.H., Lee S.-K. (2024). Leptomeningeal metastases in isocitrate dehydrogenase-wildtype glioblastomas revisited: Comprehensive analysis of incidence, risk factors, and prognosis based on post-contrast fluid-attenuated inversion recovery. Neuro-Oncology.

[26] Pohar S., Taylor W., Chandan V.S., Shah H., Sagerman R.H. (2004). Primary presentation of glioblastoma multiforme with leptomeningeal metastasis in the absence of previous craniotomy: A case report. Am. J. Clin. Oncol..

[27] Buhl R., Barth H., Hugo H.H., Hutzelmann A., Mehdorn H.M. (1998). Spinal drop metastases in recurrent glioblastoma multiforme. Acta Neurochir..

[28] Onda K., Tanaka R., Takeda N. (1986). Spinal metastases of cerebral glioblastoma: The value of computed tomographic metrizamide myelography in the diagnosis. Surg. Neurol..

[29] Alatakis S., Malham G.M., Thien C. (2001). Spinal leptomeningeal metastasis from cerebral glioblastoma multiforme presenting with radicular pain: Case report and literature review. Surg. Neurol..

[30] Arzbaecher J. (2007). Spinal metastasis in glioblastoma multiforme: A case study. J. Neurosci. Nurs..

[31] Corbett J.J., Newman N.M. (1981). Symptomatic leptomeningeal metastases preceding other manifestations of occult primary brain tumors. Surg. Neurol..

[32] Hubner F., Braun V., Richter H.P. (2001). Case reports of symptomatic metastases in four patients with primary intracranial gliomas. Acta Neurochir..

[33] Fakhrai N., Czech T., Diekmann K., Fazeny-Dorner B., Birner P., Hainfellner J.A., Prayer D., Marosi C. (2004). Glioblastoma with spinal seeding. Strahlenther. Onkol..

[34] Karaca M., Andrieu M.N., Hicsonmez A., Guney Y., Kurtman C. (2006). Cases of glioblastoma multiforme metastasizing to spinal cord. Neurol. India.

[35] Lindsay A., Holthouse D., Robbins P., Knuckey N. (2002). Spinal leptomeningeal metastases following glioblastoma multiforme treated with radiotherapy. J. Clin. Neurosci..

[36] Bukeo T., Matsumoto Y., Nishimoto A., Tabuchi K. (1985). Spinal epidural metastasis of glioblastoma multiforme: A case report. No Shinkei Geka.

[37] Chang C.C., Kuwana N., Ito S., Koike Y., Kitamura H. (2001). Spinal leptomeningeal metastases of giant cell glioblastoma associated with subarachnoid haemorrhage: Case report. J. Clin. Neurosci..

[38] Sadik A.R., Port R., Garfinkel B., Bravo J. (1984). Extracranial metastasis of cerebral glioblastoma multiforme: Case report. Neurosurgery.

[39] Lam C.H., Cosgrove G.R., Drislane F.W., Sotrel A. (1991). Spinal leptomeningeal metastasis from cerebral glioblastoma. Appearance on magnetic resonance imaging. Surg. Neurol..

[40] Kanai R., Tasaka M., Sejima H., Uchida N., Nakano A., Akiyama Y., Yamasaki T., Yamaguchi S. (2005). Brain stem glioblastoma with multiple large cyst formation and leptomeningeal dissemination in a 4-year-old girl. Brain Dev..

[41] Shahideh M., Fallah A., Munoz D.G., Loch Macdonald R. (2012). Systematic review of primary intracranial glioblastoma multiforme with symptomatic spinal metastases, with two illustrative patients. J. Clin. Neurosci..

[42] Park Y.W., Han K., Park J.E., Ahn S.S., Kim E.H., Kim J., Kang S.-G., Chang J.H., Kim S.H., Lee S.-K. (2023). Leptomeningeal metastases in glioma revisited: Incidence and molecular predictors based on postcontrast fluid-attenuated inversion recovery imaging. J. Neurosurg..

[43] Essig M., Knopp M.V., Schoenberg S.O., Hawighorst H., Wenz F., Debus J., van Kaick G. (1999). Cerebral gliomas and metastases: Assessment with contrast-enhanced fast fluid-attenuated inversion-recovery MR imaging. Radiology.

[44] Fukuoka H., Hirai T., Okuda T., Shigematsu Y., Sasao A., Kimura E., Hirano T., Yano S., Murakami R., Yamashita Y. (2010). Comparison of the added value of contrast-enhanced 3D fluid-attenuated inversion recovery and magnetization-prepared rapid acquisition of gradient echo sequences in relation to conventional postcontrast T1-weighted images for the evaluation of leptomeningeal diseases at 3T. AJNR Am. J. Neuroradiol..

[45] Ahn S.J., Taoka T., Moon W.J., Naganawa S. (2022). Contrast-Enhanced Fluid-Attenuated Inversion Recovery in Neuroimaging: A Narrative Review on Clinical Applications and Technical Advances. J. Magn. Reson. Imaging.

[46] Chen J., Shi Q., Li S., Zhao Y., Huang H. (2022). Clinical characteristics of glioblastoma with metastatic spinal dissemination. Ann. Palliat. Med..

[47] Bilgin E., Okten A.I., Duman B.B., Cavus G., Acik V., Istemen I., Altintas S. (2021). Extracranial Metastasis of IDH-1 Wild Type Glioblastomas. Turk. Neurosurg..

[48] Dardis C., Milton K., Ashby L., Shapiro W. (2014). Leptomeningeal metastases in high-grade adult glioma: Development, diagnosis, management, and outcomes in a series of 34 patients. Front. Neurol..

[49] Mistry A.M., Hale A.T., Chambless L.B., Weaver K.D., Thompson R.C., Ihrie R.A. (2017). Influence of glioblastoma contact with the lateral ventricle on survival: A meta-analysis. J. Neuro-Oncol..

[50] Wegener E., Horsley P., Wheeler H., Jayamanne D., Kastelan M., Guo L., Brown C., Back M. (2023). Leptomeningeal neuraxis relapse in glioblastoma is an uncommon but not rare event associated with poor outcome. BMC Neurol..

[51] Grabb P.A., Albright A.L., Pang D. (1992). Dissemination of supratentorial malignant gliomas via the cerebrospinal fluid in children. Neurosurgery.

[52] Mistry A.M., Kelly P.D., Gallant J.N., Mummareddy N., Mobley B.C., Thompson R.C., Chambless L.B. (2019). Comparative Analysis of Subventricular Zone Glioblastoma Contact and Ventricular Entry During Resection in Predicting Dissemination, Hydrocephalus, and Survival. Neurosurgery.

[53] Young J.S., Gogos A.J., Pereira M.P., Morshed R.A., Li J., Barkovich M.J., Hervey-Jumper S.L., Berger M.S. (2021). Effects of ventricular entry on patient outcome during glioblastoma resection. J. Neurosurg..

[54] Kato H., Fujimura M., Kumabe T., Ishioka C., Kanamaru R., Yoshimoto T. (2004). PTEN gene mutation and high MIB-1 labeling index may contribute to dissemination in patients with glioblastoma. J. Clin. Neurosci..

[55] Korshunov A., Sycheva R., Golanov A., Pronin I. (2007). Gains at the 1p36 chromosomal region are associated with symptomatic leptomeningeal dissemination of supratentorial glioblastomas. Am. J. Clin. Pathol..

[56] Shaaban A., Babu R.A., Elbadry R.G., Haddad R., Al-Bozom I., Ayyad A., Belkhair S. (2020). Spinal Metastasis of Cerebral Glioblastoma with Genetic Profile: Case Report and Review of Literature. World Neurosurg..

[57] Zhao F., Yang G., Feng M., Cao Z., Liu Y., Qiu J., You L., Zheng L., Zhang T., Zhao Y. (2020). Expression, function and clinical application of stanniocalcin-1 in cancer. J. Cell. Mol. Med..

[58] Sakata J., Sasayama T., Tanaka K., Nagashima H., Nakada M., Tanaka H., Hashimoto N., Kagawa N., Kinoshita M., Nakamizo S. (2019). MicroRNA regulating stanniocalcin-1 is a metastasis and dissemination promoting factor in glioblastoma. J. Neuro-Oncol..

[59] Andersen B.M., Miranda C., Hatzoglou V., DeAngelis L.M., Miller A.M. (2019). Leptomeningeal metastases in glioma: The Memorial Sloan Kettering Cancer Center experience. Neurology.

[60] McGirt M.J., Goldstein I.M., Chaichana K.L., Tobias M.E., Kothbauer K.F., Jallo G.I. (2008). Extent of surgical resection of malignant astrocytomas of the spinal cord: Outcome analysis of 35 patients. Neurosurgery.

[61] Minehan K.J., Shaw E.G., Scheithauer B.W., Davis D.L., Onofrio B.M. (1995). Spinal cord astrocytoma: Pathological and treatment considerations. J. Neurosurg..

[62] Lawton A.J., Lee K.A., Cheville A.L., Ferrone M.L., Rades D., Balboni T.A., Abrahm J.L. (2019). Assessment and Management of Patients With Metastatic Spinal Cord Compression: A Multidisciplinary Review. J. Clin. Oncol..

[63] Stark A.M., Nabavi A., Mehdorn H.M., Blomer U. (2005). Glioblastoma multiforme-report of 267 cases treated at a single institution. Surg. Neurol..

[64] Jahraus C.D., Dishop M.K., Bayliff S.L., Lee C., St Clair W.H. (2003). Atypical presentation and progression of glioblastoma multiforme in a 6-year-old girl: Multidisciplinary case report. J. Pediatr. Hematol. Oncol..

[65] Liu J., Zheng M., Yang W., Lo S.L., Huang J. (2018). Impact of surgery and radiation therapy on spinal high-grade gliomas: A population-based study. J. Neuro-Oncol..

[66] Scoccianti S., Detti B., Meattini I., Iannalfi A., Sardaro A., Leonulli B.G., Martinelli F., Bordi L., Pellicano G., Biti G. (2008). Symptomatic leptomeningeal and intramedullary metastases from intracranial glioblastoma multiforme: A case report. Tumori.

[67] Fiorentino A., Caivano R., Chiumento C., Cozzolino M., Fusco V. (2012). Radiotherapy and bevacizumab for intramedullary and leptomenigeal metastatic glioblastoma: A case report and review of the literature. Int. J. Neurosci..

[68] Shah A., Redhu R., Nadkarni T., Goel A. (2010). Supratentorial glioblastoma multiforme with spinal metastases. J. Craniovertebral Junction Spine.

[69] Grah J.J., Katalinic D., Stern-Padovan R., Paladino J., Santek F., Juretic A., Zarkovic K., Plestina S., Supe M. (2013). Leptomeningeal and intramedullary metastases of glioblastoma multiforme in a patient reoperated during adjuvant radiochemotherapy. World J. Surg. Oncol..

[70] Linsenmann T., Monoranu C.M., Vince G.H., Westermaier T., Hagemann C., Kessler A.F., Ernestus R.I., Löhr M. (2014). Long-term tumor control of spinal dissemination of cerebellar glioblastoma multiforme by combined adjuvant bevacizumab antibody therapy: A case report. BMC Res. Notes.

[71] Kirkpatrick J.P., van der Kogel A.J., Schultheiss T.E. (2010). Radiation dose-volume effects in the spinal cord. Int. J. Radiat. Oncol. Biol. Phys..

[72] Sahgal A., Chang J.H., Ma L., Marks L.B., Milano M.T., Medin P., Niemierko A., Soltys S.G., Tomé W.A., Wong C.S. (2021). Spinal Cord Dose Tolerance to Stereotactic Body Radiation Therapy. Int. J. Radiat. Oncol. Biol. Phys..

[73] Lazzari G., Montagna A., Benevento I., D’Andrea B., Metallo V., Tucciariello R., Colamaria A., Di Perna G., Modano P., Bianculli A. (2025). Safety and Efficacy of Stereotactic Radiosurgery in the Management of Primary Spinal Cord Glioblastoma: A Case Report. Cancer Manag. Res..

[74] National Comprehensive Cancer Network NCCN Guidelines: Central Nervous System Cancers 2024 (Version 5). https://www.nccn.org/guidelines/guidelines-detail?category=1&id=1425.

[75] Weller M., van den Bent M., Preusser M., Le Rhun E., Tonn J.C., Minniti G., Bendszus M., Balana C., Chinot O., Dirven L. (2021). EANO guidelines on the diagnosis and treatment of diffuse gliomas of adulthood. Nat. Rev. Clin. Oncol..

[76] Stupp R., Brada M., van den Bent M.J., Tonn J.C., Pentheroudakis G. (2014). High-grade glioma: ESMO Clinical Practice Guidelines for diagnosis, treatment and follow-up. Ann. Oncol..

[77] Fazzari F.G.T., Rose F., Pauls M., Guay E., Ibrahim M.F.K., Basulaiman B., Tu M., Hutton B., Nicholas G., Ng T.L. (2022). The current landscape of systemic therapy for recurrent glioblastoma: A systematic review of randomized-controlled trials. Crit. Rev. Oncol./Hematol..

[78] Burger M.C., Zeiner P.S., Jahnke K., Wagner M., Mittelbronn M., Steinbach J.P. (2016). Addition of Anti-Angiogenetic Therapy with Bevacizumab to Chemo- and Radiotherapy for Leptomeningeal Metastases in Primary Brain Tumors. PLoS ONE.

[79] Birzu C., Tran S., Bielle F., Touat M., Mokhtari K., Younan N., Psimaras D., Hoang-Xuan K., Sanson M., Delattre J.Y. (2020). Leptomeningeal Spread in Glioblastoma: Diagnostic and Therapeutic Challenges. Oncologist.

[80] Louis D.N., Perry A., Wesseling P., Brat D.J., Cree I.A., Figarella-Branger D., Hawkins C., Ng H.K., Pfister S.M., Reifenberger G. (2021). The 2021 WHO Classification of Tumors of the Central Nervous System: A summary. Neuro-Oncology.

