# Supplementary material for: Spinal Metastasis from Supratentorial Glioblastoma: A Registry-Based Case Series and a Review of the Literature
Source: Cancers (Basel). 2025 Sep 12;17(18):2979. doi: 10.3390/cancers17182979 (PMC12468205; doi:10.3390/cancers17182979)
Supplement: Supplementary file 1 [file cancers-17-02979-s001.zip › cancers-3746563-supplementary.pdf]

cancers MDPI

Table S1. Characteristics of the three patients with spinal metastasis at the time of the diagnosis (i.e. ≤ 1 month of the index operation).

	Time between index operation and detection of spinal metastasis	Distribution of the spinal metastatic lesions	Treatment offered	Post-spinal metastasis survival
Patient 10 (44/M; bilateral frontal lobe; *IDH*-1 mutation and p*MGMT* methylation status not available)	26 days	Diffuse involvement from cranio-cervical junction to T9 level, and L2-S1	Tumour biopsy of the frontal tumor<br>Palliative craniospinal radiotherapy (36 Gy), with boost to the bilateral frontal lobe lesions (to 54 Gy) and C5-T1/ L1-S2 spinal lesions (to 45 Gy)	14.6 weeks
Patient 12 (48/F; left temporal lobe; *IDH*-1 mutation and p*MGMT* methylation status not available)	12 days	T3, T5, and T7-10	Subtotal resection of the temporal tumor<br>Dabrafenib + trametinib	15.4 weeks
Patient 14 (22/F; right temporal lobe; *IDH*-1 wildtype and p*MGMT* unmethylated)	19 days	Diffuse leptomeningeal spread along the whole spine	Subtotal resection of the temporal tumor<br>TMZ → dabrafenib + trametinib with subsequent nivolumab<br>Palliative radiotherapy to C1-T7 (15 Gy in 5 fractions), and later to T8 - sacrum (single fraction) for pain control	28.7 weeks

N.B. IDH-1, isocitrate dehydrogenase-1; p*MGMT*, promoter region of the methylguanine methyl transferase gene; TMZ, temozolomide.
